# Filling in the gap: two new records and an updated distribution map for the Gulf Sand gecko *Pseudoceramodactylus khobarensis* Haas, 1957

**DOI:** 10.3897/BDJ.2.e4011

**Published:** 2014-10-06

**Authors:** Margarita Metallinou, Raquel Vasconcelos, Jiří Šmíd, Roberto Sindaco, Salvador Carranza

**Affiliations:** † Institute of Evolutionary Biology (CSIC-Universitat Pompeu Fabra), Barcelona, Spain; ‡ CIBIO, Centro de Investigação em Biodiversidade e Recursos Genéticos, InBIO Laboratório Associado, Universidade do Porto, Vairão, Portugal; § Department of Zoology, National Museum, Prague, Czech Republic; | Department of Zoology, Faculty of Science, Charles University in Prague, Prague, Czech Republic; ¶ Museo Civico di Storia Naturale, Carmagnola (TO), Italy

**Keywords:** Reptilia, Gekkonidae, DNA, 12S, distribution range, Arabia, sabkha

## Introduction

The genus *Pseudoceramodactylus* Haas, 1957 comprises a single species, the Gulf Sand gecko *Pseudoceramodactylus khobarensis*, described from eastern Saudi Arabia (Haas 1957) and is known to be distributed across parts of the Arabian Gulf, including Kuwait, Bahrain, Qatar and the United Arab Emirates (U.A.E.) (Sindaco and Jeremčenko 2008, Valdeón et al. 2013). It has also been reported from Qeshm Island, Iran (Dakhteh et al. 2007, Sharifi et al. 2012) and a few localities are known from coastal eastern Oman (Fujita and Papenfuss 2011, Gardner 2013, Metallinou et al. 2012). *Pseudoceramodactylus khobarensis* are nocturnal geckos, found on moist, salt-impregnated to solid, salt-encrusted flats (sabkhas) (Fig. 1a, b, c) and are often the sole reptile dweller of such extreme environments (Arnold 1977, Gardner 2013). Their fingers are swollen with loose connective tissue and bear numerous elongated spiny scales on the underside (Arnold 1977), considered to be an adaptation to this particular substrate.

*Pseudoceramodactylus khobarensis* was transferred to the genus *Stenodactylus* by Kluge (1967) on the basis of external and internal similarities. Nevertheless, authors recognized its singularity among the other *Stenodactylus* members, due to its remarkably swollen nasal area, enlarged postmental scales and slender, elongated extremities (Arnold 1980). Using molecular data, Fujita and Papenfuss (2011) showed that its inclusion in *Stenodactylus* rendered the latter paraphyletic, so the genus *Pseudoceramodactylus* was resurrected. Metallinou et al. (2012) confirmed this result but, performing topological tests, they showed that the sister relationship between *Pseudoceramodactylus* and *Stenodactylus* could not be rejected. The same authors included specimens from the two extremes of the species’ range – Kuwait and Oman – in their study, and found only small genetic divergence in mitochondrial DNA (12S and 16S rRNA markers) between specimens from these areas (see Additional file 2 – Figure S1 in Metallinou et al. 2012). However, according to the known range, localities of *Pseudoceramodactylus khobarensis* in coastal Oman are isolated and separated by more than 420 km from the eastern localities in inland U.A.E.

**Figure 2.**
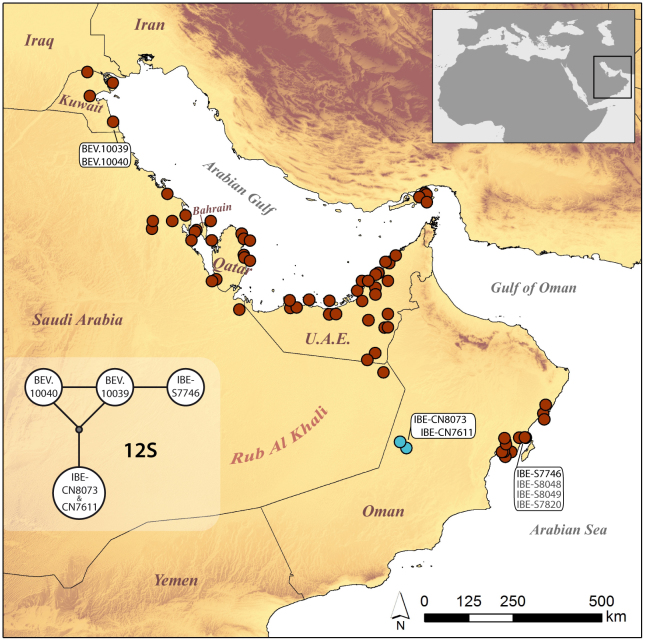
Updated distribution map for *Pseudoceramodactylus khobarensis*, with new records in light blue color. Localities of material examined in this study are indicated and corresponding specimen codes are provided. In the inset figure, the haplotype network of the mitochondrial marker 12S is presented. Exact localities, 12S GenBank accession codes and morphological measurements of specimens are listed in Table 2.

Herein, we report two new records for this species from the eastern edge of the Rub Al Khali desert (‘Uruq al Mu’taridah area), in inner Oman (Fig. 2), indicating that this distributional gap is rather attributed to incomplete knowledge of the species’ distribution than actual absence. Morphological data and mitochondrial DNA (mtDNA) analyses are presented and the distribution and biogeography of this monotypic genus are briefly discussed.

## Materials and methods

During intensive fieldwork in Oman, in October 2013, we surveyed the easternmost tip of the Rub Al Khali desert (Fig. 2). Individual transects were carried out by five observers and collecting was conducted manually. We collected two specimens of *Pseudoceramodactylus khobarensis* in two different localities near the border between Oman and Saudi Arabia, in an area of salt-encrusted flats and interdune sabkha (Table 1 and Fig. 1c). The two vouchers collected are housed at Salvador Carranza’s reptile collection at the Institute of Evolutionary Biology, Barcelona, Spain.

Data for the updated distribution map were compiled from Gallagher (1971), Osborne (1994), Martens (1996), Meinig and Kessler (1998), Cunningham (2000), Dakhteh et al. (2007), Fujita and Papenfuss (2011), Gardner (2013), Valdeón et al. (2013). The map was produced by representing coordinates from literature records and by georeferencing figures and extracting point coordinates with ArcGIS 10.0 (ESRI 2010).

A total of five individuals, the two newly collected ones and three additional specimens from the extremes of the species’ range (Fig. 2), were analyzed for variation in the mtDNA. Genomic DNA was extracted from ethanol-preserved tongue tissue samples from the newly collected specimens using the SpeedTools Tissue DNA Extraction kit (Biotools, Madrid, Spain). The mtDNA marker 12S rRNA gene was partially amplified using primers and conditions from Metallinou et al. (2012). Amplified fragments were sequenced for both strands and chromatograph contigs were assembled in Geneious v. R6 (Biomatters Ltd.). The online version of MAFFT v.6 (Katoh and Toh 2008) was used for sequence alignment, applying parameters by default. A median-joining haplotype network was constructed using the Fluxus Phylogenetic Network Analysis software v.4.612 (Bandelt et al. 1999; http://www.fluxus-engineering.com). Uncorrected *p*-distances between individuals were calculated with MEGA 5 (Tamura et al. 2011).

A series of morphological measurements were performed on the same five individuals, as well as three additional specimens from the locality in coastal Oman belonging to the field series of S. Carranza (Table 2 and Fig. 2) Measurements were taken by the first author on the right side of each specimen (unless defective), using a digital caliper with accuracy to the nearest 0.01 mm. Specimens were sexed by observing presence or absence of hemipenal bulges in adult specimens and measurements were performed as follows: snout-vent length (SVL) measured from tip of snout to vent; head length (HL), measured tip of snout to posterior ear opening border; head width (HW), measured dorsally as the distance between the eyes excluding the eyelid; transverse eye diameter (ED); forearm length (FL), from base of palm to elbow; arm length (AL), from elbow to the insertion of the forelimb on the posterior side; tibia length (BL), measured from base of foot to knee; femur length (ML), measured from knee to the insertion of the hind limb on the posterior side; tail length (TL), from vent to tip of tail; number of upper labial scales (ULS) and number of lower labial scales (LLS).

## Taxon treatments

### Pseudoceramodactylus khobarensis

Haas, 1957

http://reptile-database.reptarium.cz/species?genus=Pseudoceramodactylus&species=khobarensis

http://eol.org/pages/461035/overview

http://www.itis.gov/servlet/SingleRpt/SingleRpt?search_topic=TSN&search_value=819426

#### Materials

- **Type status:** Other material. **Occurrence:** recordedBy: Salvador Carranza; Raquel Vasconcelos; Margarita Metallinou; Roberto Sindaco; Jiri Smid; individualCount: 1; sex: male; **Taxon:** taxonID: http://www.gbif.org/species/2447065#; scientificNameID: urn:lsid:organismnames.com:name:2791139; **Location:** country: Oman; stateProvince: Al Wusta; verbatimLocality: north of Hasirah oil field, ‘Uruq al Mu’taridah area; verbatimElevation: 143 m; verbatimLatitude: 20 30 7.704N; verbatimLongitude: 55 41 56.2554E; **Event:** eventDate: 2013-10-07T00:30+0400; **Record Level:** collectionID: IBE-CN7611; institutionCode: Institute of Evolutionary Biology (CSIC - Universitat Pompeu Fabra)
- **Type status:** Other material. **Occurrence:** recordedBy: Salvador Carranza; Roberto Sindaco; Margarita Metallinou; Raquel Vasconcelos; Jiri Smid; individualCount: 1; sex: juvenile; **Taxon:** taxonID: http://www.gbif.org/species/2447065#; scientificNameID: urn:lsid:organismnames.com:name:2791139; **Location:** country: Oman; stateProvince: Al Wusta; verbatimLocality: about 13km by air east of Sahmah oil filed, ‘Uruq al Mu’taridah area; verbatimElevation: 96 m; verbatimLatitude: 20 39 37.0434N; verbatimLongitude: 55 32 28.716E; **Event:** eventDate: 2013-10-07T02:00+0400; **Record Level:** collectionID: IBE-CN8073; institutionCode: Institute of Evolutionary Biology (CSIC - Universitat Pompeu Fabra)

## Analysis

Analysis of the mitochondrial 12S marker revealed that both newly collected specimens share the same haplotype. Along a 380-bp alignment, there are 3 differences compared to sample IBE-S7746 from Barr Al-Hickman, in coastal Oman (M196 in Metallinou et al. 2012) (0.6% *p*-distance) and 2 differences compared to either one from Kuwait BEV.10039 and BEV.10040 (M16 and M37 in Metallinou et al. 2012, respectively) (0.3% *p*-distance). The overall genetic variability based on this marker was 0.4%.

The mean SVL of the specimens measured was 53.87 mm (50.71–61.37, N=7), and did not differ for males (53.92, 51.71–61.37, N=5) and females (53.76, 50.72–56.80, N=2). Tail length measured between 71.1 and 95.1% of SVL.

## Discussion

The Rub Al Khali is the largest desert in Arabia, the largest sand desert in the world and one of the driest (Garzanti et al. 2003, Vincent 2008). It extends across Saudi Arabia with its southern and eastern edges reaching Yemen, Oman and the U.A.E. In Oman, accessibility to this region is limited, and this is mirrored in the paucity of reptile records available, contrasting with the more abundant records from the U.A.E. in areas of similar ecological characteristics (Gardner 2013). We surveyed the easternmost tip of this desert in Oman on the night of 7 October 2013 and we collected two specimens of *Pseudoceramodactylus khobarensis* in two different localities near the border between Oman and Saudi Arabia (Fig. 2 and Table 1): a male specimen (voucher code IBE-CN7611) (Fig. 1a, b) north of Hasirah oil field, and a juvenile specimen (IBE-CN8073) about 13 km by air east of Sahmah oil filed. In the first locality, the habitat was exclusively salt-encrusted flats, and *Pseudoceramodactylus khobarensis* was the only species encountered during a 40-minute survey carried out by 5 observers. In the second locality, there was a succession between salt flats and sand dunes (Fig. 1c), with *Pseudoceramodactylus khobarensis* found on the former and *Stenodactylus arabicus* on the latter. These records constitute the first inland records of *Pseudoceramodactylus khobarensis* from Oman and are located almost 250 km from both the eastern records in inland U.A.E. and those in eastern coastal Oman (Fig. 2). The finding of *Pseudoceramodactylus khobarensis* in this area indicates that its presence in Oman is most probably underestimated, due to the aforementioned difficulty of access to large parts of the inland deserts.

Interestingly, the low variability of the mtDNA observed with the sequenced marker (12S) indicates that there is probably connectivity between populations across its distribution range and corroborates the hypothesis that this species inhabits larger inland areas. Indeed, coastal and inland sabkhas are abundant in eastern Saudi Arabia (Barth 2002) and continental sabkhas are commonly found at the interdune corridors of north-eastern Rub Al Khali (Edgell 2006). This low variability observed in *Pseudoceramodactylus khobarensis* contrasts with the much higher values observed in some members of the closely related genus *Stenodactylus*, as calculated based on specimens distributed across similar ranges in Metallinou et al. (2012) and Metallinou and Carranza (2013). Intraspecific variability ranged from 0.9% in *Stenodactylus leptocosymbotes*, to 1.6% in *Stenodactylus arabicus* and 2.9% in *Stenodactylus doriae*.

Based on the measurements performed on voucher specimens in this study, *Pseudoceramodactylus khobarensis* is shown to have substantially higher maximum SVL than previously documented. One female reached 56.80 mm (BEV.10040) and one male 61.37 mm (IBE-CN7611), both exceeding SVL of the largest specimens measured by Haas (1957) and Arnold (1980). Moreover, numbers of labial scales presented herein (upper 10–13, lower 9–12) seem to be slightly different from the counts given for the type series by Haas (1957) (9/10 upper, 8/9 lower labials) but are in perfect agreement with those by Arnold (1980) who included one specimen from the original description in his examined material. Therefore, this situation can probably be attributed to observer-related discrepancies, rather than an actual difference in counts of this meristic variable.

*Pseudoceramodactylus khobarensis* is a remarkable desert reptile in that it is the only lizard habitually found on sabkha substrate (Arnold 1977), a habitat almost devoid of vegetation due to extraordinary salinity (König 2012). The species is widespread and classified by the International Union for Conservation of Nature (IUCN) as Least Concern, but a decreasing population trend is observed in parts of its range due to ongoing significant habitat loss through coastal development, especially in the U.A.E. (Sharifi et al. 2012). In this way, it is important to document the species’ distribution and understand its ecological requirements at national and regional scales in order to prevent imperilment in larger parts of its range.

## Supplementary Material

XML Treatment for Pseudoceramodactylus khobarensis

## Figures and Tables

**Figure 1a.**
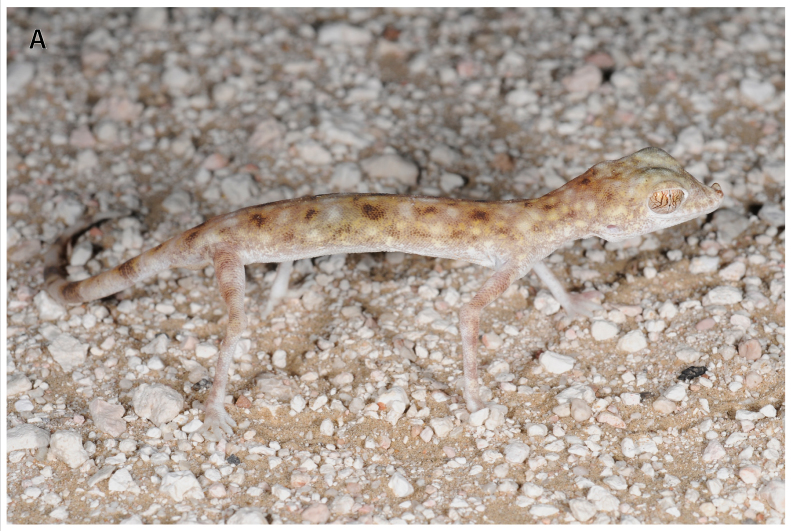
Male specimen of *Pseudoceramodactylus khobarensis* (IBE-CN7611) from eastern Rub Al Khali desert in Oman, in life, presenting the singular elongated extremities.

**Figure 1b.**
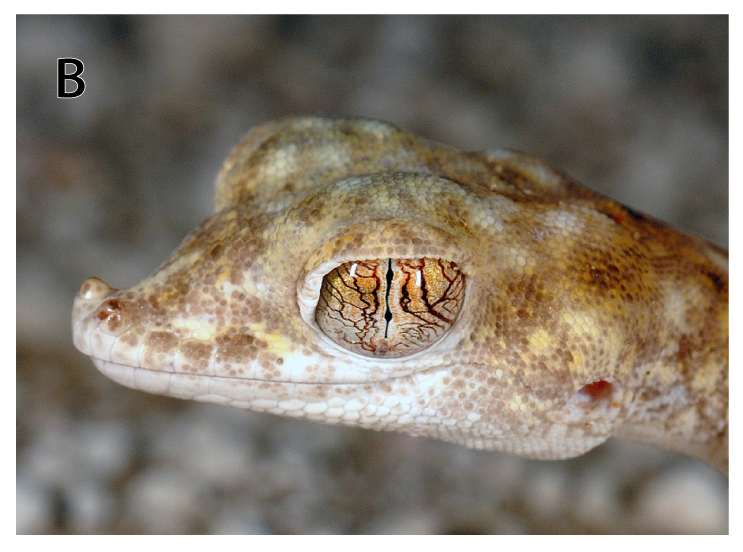
Detail of the left side of the head of the same specimen, where it is possible to observe the swollen nasal area.

**Figure 1c.**
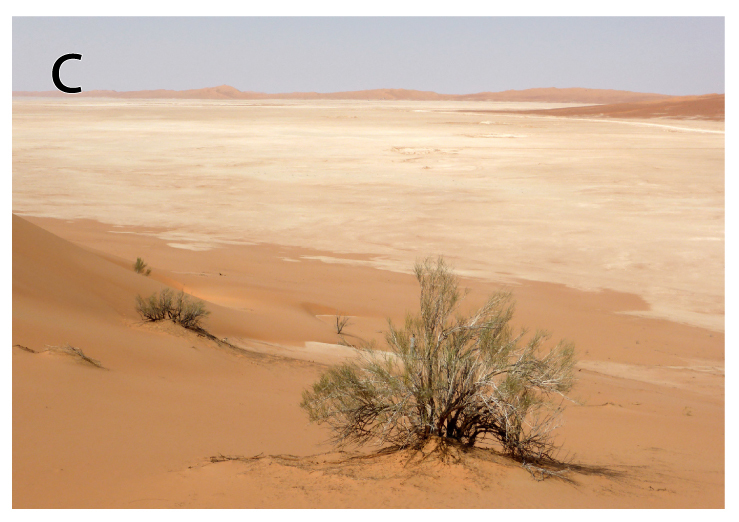
Interdune sabkha in the general area where a juvenile *Pseudoceramodactylus khobarensis* was collected, north of Hasirah oil field, in central-western Oman. Both specimens were found active during the night (Table 1), on salt-encrusted substrate.

**Table 1.** New records of localities where *Pseudoceramodactylus khobarensis* was collected in eastern Rub Ak Khali desert, Oman.

Specimen Code	Date	Time	Latitude	Longitude	Elevation (m)	Temperature (°C)	Humidity	Other species collected
IBE-CN8073	7-Oct-2013	02:00 am	20.66029	55.54131	96	27.8	50.2	*Stenodactylus arabicus*
IBE-CN7611	7-Oct-2013	00:30 am	20.50214	55.69896	143	28.3	57.6	none

**Table 2.** Morphometrical (in mm) and meristic measurements for the specimens examined in this study, originating from four different localities across the range of *Pseudoceramodactylus khobarensis* (see Fig. 2).

Specimen Code	IBE-CN8073	IBE-CN7611	IBE-S7746	IBE-S8048	IBE-S8049	IBE-S7820	BEV.10039	BEV.10040
GenBank 12S	KM047415	KM047415	KC190704	-	-	-	KC190703	KC190702
Latitude	20.6603	20.5021	20.6854	20.6854	20.6854	20.6854	28.6369	28.6369
Longitude	55.5413	55.699	58.2934	58.2934	58.2934	58.2934	48.1336	48.1336
Sex	(juvenile)	male	male	male	male	female	male	female
Snout-vent length (SVL)	28.52	61.37	50.90	53.56	53.05	50.72	50.71	56.80
Head length (HL)	8.28	15.08	13.17	14.34	14.69	13.00	13.29	14.50
Head width (HW)	4.78	7.93	6.17	6.56	6.93	6.21	7.25	7.80
Eye diameter (ED)	2.60	4.28	4.02	3.74	4.07	4.15	3.63	4.17
Forearm length (FL)	7.72	12.61	11.36	10.96	11.48	11.64	11.37	11.59
Arm length (AL)	4.54	9.19	7.32	7.62	8.24	7.78	7.76	8.10
Tibia length (BL)	7.64	13.79	12.39	11.91	13.43	13.05	11.55	13.68
Femur length (ML)	6.66	13.63	10.50	11.54	13.32	11.86	10.61	12.10
Tail length (TL)	27.13	53.74	36.19	43.56	40.04	38.76	N/A	47.45
Upper labials (ULS)	13	13	12	12	11	12	12	12
Lower labials (LLS)	10	12	10	11	10	11	12	11

## References

[B740481] Arnold E. N. (1977). Little-known geckoes (Reptilia: Gekkonidae) from Arabia with descriptions of two new species from the Sultanate of Oman. The Scientific Results of the Oman Flora and Fauna Survey 1975.

[B740491] Arnold E. N. (1980). Reptiles of Saudi Arabia: A Review of the Lizard Genus *Stenodactylus* (Reptilia: Gekkonidae). Fauna of Saudia Arabia.

[B740501] Bandelt Hans-Jurgen, Forster Peter, Röhl Arne (1999). Median-joining networks for inferring intraspecific phylogenies. Molecular biology and evolution.

[B841952] Barth Hans-Joerg, Barth Hans-Joerg, Böer Benno (2002). The sabkhat of Saudi Arabia - An Introduction. Sabkha Ecosystems: Volume I: The Arabian Peninsula and Adjacent Countries.

[B764357] Cunningham P. (2000). Notebook. Reptile Records. Bulletin of the Emirates Natural History Group.

[B740511] Dakhteh Seyyed Mohammad Hashem, Kami Haji Gholi, Anderson Steven C (2007). *Stenodactylus
khobarensis* (Haas, 1957): An addition to the Iranian herpetofauna (Reptilia: Squamata: Gekkonidae). Russian Journal of Herpetology.

[B841966] Edgell H. Stewart (2006). Arabian Deserts: Nature, Origin and Evolution.

[B764387] ESRI (2010). Arcmap 10. Environmental Systems Research Institute.

[B740521] Fujita M. K., Papenfuss T. J. (2011). Molecular systematics of *Stenodactylus* (Gekkonidae), an Afro-Arabian gecko species complex. Molecular phylogenetics and evolution.

[B764273] Gallagher M. D. (1971). The Amphibians and Reptiles of Bahrain. British Museum (Natural history).

[B740531] Gardner Andrew S (2013). The Amphibians and Reptiles of Oman and the UAE.

[B740540] Garzanti Eduardo, Andò Sergio, Vezzoli Giovanni, Dell'era Daniela (2003). From Rifted Margins to Foreland Basins: Investigating Provenance and Sediment Dispersal Across Desert Arabia (Oman, U.A.E.). Journal of Sedimentary Research.

[B740696] Haas G. (1957). Some amphibians and reptiles from Arabia. Proceedings of the California Academy of Sciences.

[B740550] Katoh K., Toh H. (2008). Recent developments in the MAFFT multiple sequence alignment program. Briefings in bioinformatics.

[B740560] Kluge A. G. (1967). Higher taxonomic categories of gekkonid lizards and their evolution. Bulletin of the American Museum of Natural History.

[B741628] König P. (2012). Plant life in the Umm as Samim, Oman – A case study in a major inland sabkha. Journal of Arid Environments.

[B789768] Martens H., Krupp F., Abuzinada A. H., Nader I. A. (1996). A preliminary survey of the terrestrial reptile and sea snakes in the Jubail Marine Wildlife Sanctuary. A marine wildlife sanctuary for the Arabian Gulf: environmental research and conservation following the 1991 Gulf War Oil Spill.

[B764337] Meinig H., Kessler H. (1998). Herpetological observations within the framework of national park planning: Barr al Hikman and Masirah Island, Sultanate of Oman (Amphibia, Reptilia).. Faunistische Abhandlungen (Dresden).

[B740586] Metallinou Margarita, Carranza Salvador (2013). New species of *Stenodactylus* (Squamata: Gekkonidae) from the Sharqiyah Sands in northeastern Oman. Zootaxa.

[B740570] Metallinou M., Arnold E. N., Crochet P. -A., Geniez P., Brito J. C., Lymberakis P., Baha El Din S., Sindaco R., Robinson M., Carranza S. (2012). Conquering the Sahara and Arabian deserts: Systematics and biogeography of *Stenodactylus* geckos (Reptilia: Gekkonidae). BMC Evolutionary Biology.

[B764327] Osborne P. (1994). Reptiles. Tribulus.

[B789758] Sharifi M., Papenfuss T., Els J., Shafiei Bafti S., Al Johany A. M.H. (2012). *Pseudoceramodactylus
khobarensis*. In: IUCN 2014. IUCN Red List of Threatened Species. http://www.iucnredlist.org/details/164699/0.

[B740596] Sindaco R., Jeremčenko V. K. (2008). The reptiles of the Western Palearctic.

[B740605] Tamura K., Peterson D., Peterson N., Stecher G., Nei M., Kumar S. (2011). MEGA5: molecular evolutionary genetics analysis using maximum likelihood, evolutionary distance, and maximum parsimony methods. Molecular biology and evolution.

[B740617] Valdeón Aitor, Castilla Aurora M, Cogalniceanu Dan, Gosá Alberto, Alkuwary Ali, Saifelnasr E. O.H., Naumann Elsa, Mas-Peinado Paloma, Richer Renee, Al-Hemaidi Ahmad Amer Mohd (2013). On the presence and distribution of the Gulf sand gecko, *Pseudoceramodactylus
khobarensis* Haas, 1957 (Reptilia: Squamata: Gekkonidae) in Qatar. QScience Connect.

[B740816] Vincent Peter (2008). Saudi Arabia: An environmental review.

